# Image-guided radiation therapy using surgical clips for localization of colonic metastasis from thyroid cancer

**DOI:** 10.1186/s13014-014-0298-z

**Published:** 2014-12-24

**Authors:** Alvin Szeto, Lee Chin, Patrick Whelan, Jennifer Wilson, Justin Lee

**Affiliations:** Department of Radiation Oncology, Sunnybrook Odette Cancer Centre, University of Toronto, 2075 Bayview Avenue, Toronto, ON M4N 3M5 Canada; Department of Surgery, Markham Stouffville Hospital, Markham, Ontario Canada; Department of Family and Community Medicine, Markham Stouffville Hospital, Markham, Ontario Canada

**Keywords:** Fiducial markers, Image-guided radiation therapy, Surgical clips, Thyroid cancer, Colonic metastasis, Lower gastrointestinal bleed

## Abstract

A 67-year old man with a history of papillary thyroid cancer (PTC) presented with metastatic disease to the left colon in the form of a 6.1x1.0 cm bleeding, ulcerated mass. Radiopaque surgical clips were used as fiducial markers to localize the gross tumor volume (GTV) as well as the corresponding clinical target volume (CTV) and planning target volume (PTV). Daily cone beam computed tomography (CBCT) image guidance was utilized to verify the tumor position. Inter- and intrafraction movement of the tumor mass was assessed. Gastrointestinal bleeding was controlled using palliative image-guided radiation therapy (IGRT).

## Background

Papillary thyroid cancer (PTC) is the most common form of well differentiated thyroid cancer and is usually associated with good prognosis [1]. Recurrence rates are reported between 10-33% depending on stage and histological subtype [2]. Common sites for distant metastases of PTC include mediastinal lymph nodes, lung and bone [3]. Here, we discuss a patient with recurrent PTC with distant metastases to mediastinal lymph nodes, lung, spine, as well as colon. Gastrointestinal metastases of thyroid cancer account for 0.5-1% metastatic sites [3]. To date, there are only a small number of patient reports documenting metastatic disease from the thyroid to the colon [4-6].

Radiation treatment of tumors of the colon is not typically performed as it presents a challenge to visualize and consistently target mobile abdominal organs. Treatment planning without adequate visualization of the target area can result in wide treatment margins and excessive toxicity. Movement of the intra-abdominal organs can result in underdosing of the target or overdosing of the surrounding organs. Furthermore, conventional CBCT techniques rely on bony matching, which does not necessarily correlate with internal organ motion. Use of fiducial markers, such as surgical clips, can narrow the treatment volume and improve radiation targeting. Previous studies demonstrate that use of surgical clips for targeting prostate cancer, results in a reduction in total setup error, smaller planning target volume (PTV) margin and rectal sparing [7]. Despite these potential benefits, fiducial markers may also have a negative impact on clinical outcomes if the PTV margin is significantly reduced without careful consideration of intrafraction organ motion. [8].

Here, we present a unique case of multi-site metastasis of papillary thyroid carcinoma including an ulcerated, bleeding colonic mass. Furthermore, we describe our experience with image-guided radiation therapy (IGRT) for palliative treatment to control bleeding of metastatic disease to the colon.

## Case presentation

A 67-year old man presented with an ulcerated, bleeding mass in the colon, shown by biopsy to be metastatic PTC in May 2014.

He initially presented with a locally advanced thyroid tumour in 2005 treated with total thyroidectomy and bilateral neck dissection. Post-surgical pathology revealed the primary lesion to be classic papillary thyroid carcinoma with areas of follicular and focal oncocytic variants. There was evidence of capsular invasion, extra-thyroidal extension involving muscle, adherence to the trachea and involved neck nodes; tumour stage was pT4N2M0. The patient underwent adjuvant radioactive iodine, and external beam radiation to a dose of 60Gy over 30 fractions to the thyroid bed and neck.

In June 2007, the patient developed locoregional recurrence which was treated with left neck dissection and radioactive iodine of 150 millicuries. In December 2009, chest imaging revealed pulmonary and mediastinal metastases which were treated with a third course of radioactive iodine of 157 millicuries. In January 2013, the patient presented with spinal metastases involving the T9, T11, and L2 vertebrae encroaching on the spinal canal. This was treated with radiation dose of 20Gy in 5 fractions to control neuropathy and bony pain.

In June 2014, the patient presented with abdominal fullness and lower gastrointestinal bleeding. Abdominal and pelvic CT with contrast showed metastatic deposits throughout the abdomen and pelvis, with lymphadenopathy involving the gastrohepatic ligament, celiac axis, aortocaval and left external iliac regions. The patient was found to have a hemoglobin (HgB) level of 65 mg/L and was transfused a total of 4 units of packed red blood cells.

Colonoscopy was performed; an actively bleeding 6.1 × 1.0 cm ulcerated lesion was dominant in the left colon. Biopsy of the colon mass was performed and the immunohistochemical profile was positive for TTF-1 and CK7, negative for CDX2, CK20 and HBME1. Immunohistochemistry and morphology were consistent with metastatic papillary thyroid cancer. Further surgical management of the mass was not deemed appropriate given the patient’s extent of disease and comorbidities. Additional radioactive iodine treatment was not recommended after considering the three previous doses and the low likelihood of targeting the colonic lesion. Therefore, the patient was planned for radiotherapy with the intent to stop bleeding from the colonic mass. A palliative dose of 20 Gy in 5 fractions was chosen to try to alleviate symptoms while limiting toxicity to the adjacent, unaffected organs at risk.

### Colon tumor localization

**Figure 1:**
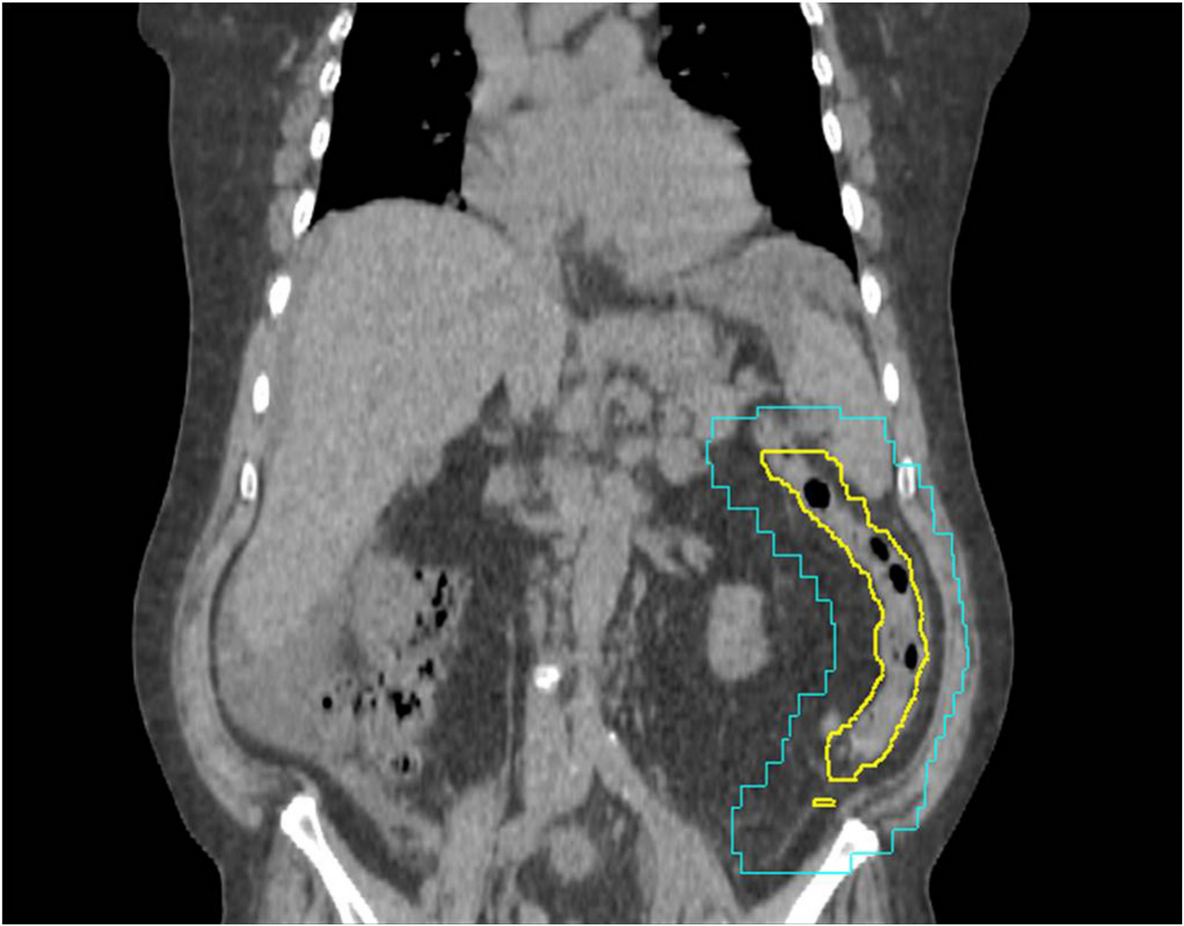
**Computed tomography of palliative radiotherapy plan without clips.** Representative coronal CT image of the CTV (yellow) and radiation field edges (blue) without fiducial markers present. No GTV was visible therefore the CTV contour included the entire descending colon.

**Figure 2:**
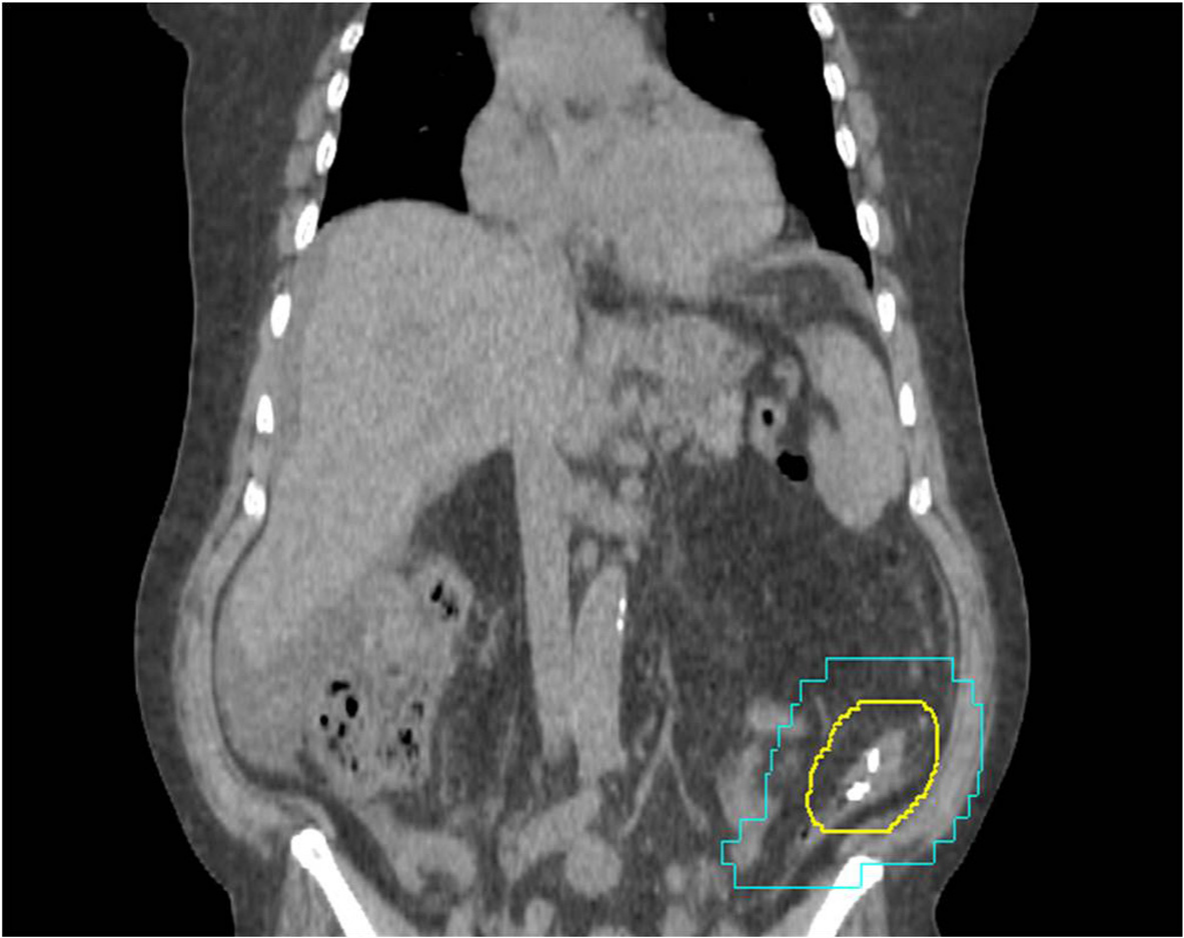
**Computed tomography of palliative radiotherapy plan with clips placed.** Representative coronal CT image of the CTV (yellow) and radiation field edges (blue). Three clips were placed within the tumour and at the distal and proximal extent. The PTV volume was decreased from 393.8 cm^3^ to 251.5 cm^3^. The V15 for small bowel loops was reduced from 328.0 cm^3^ to 176.0 cm^3^.

The initial colonoscopy described the location of the mass to be distal to the splenic flexure. However, the mass could not be resolved on diagnostic CT with contrast or initial CT simulation. Due to this uncertainty, the initial radiation treatment CTV and PTV encompassed the entire descending colon (Figure 1). It was felt that this large irradiated volume might cause excessive acute gastrointestinal side effects. Therefore, to better localize the colonic metastasis, three metallic surgical clips were inserted during repeat colonoscopy: proximal, distal and one within the ulcerated mass. On re-simulation, the surgical clips were found to be in the left lower quadrant just proximal to the sigmoid colon. The GTV was contoured to encompass the three clips and the segment of colon defined by the ends of the clips. Due to uncertainties regarding the extent of disease and intrafraction organ motion, a volumetric CTV expansion of 1 cm was applied followed by an additional PTV margin of 1 cm. Localization with the clips reduced the PTV from 393.8 cm^3^ to 251.5 cm^3^ while shifting the geometric center of the PTV by 13.7 cm (Figure 2).

### Clinical treatment technique: image guided radiotherapy with colon fiducial markers

Simulation and treatment were performed with the patient in the supine position, without contrast, using a knee support for comfort. Bladder filling was not specified for the simulation or treatment.

On treatment, daily CBCT verification was performed using image fusion from the Pinnacle 9.2 Treatment Planning System (TPS; Philips Healthcare, Andover, MA) to ensure the PTV encompassed the three clips and CTV contour. Bony registration was first confirmed on preliminary CBCT and then, if required, any additional shifts were applied to ensure that all three clips were encompassed by the PTV volume. The three clips were well visualized by radiation therapy staff and centered within the PTV volume on each of the five treatment days.

### Analysis of inter- and intrafractional organ movement

A review of each daily CBCT image and treatment shifts was performed to determine tumour motion using the surgical clips as a surrogate to define the GTV. The clips were contoured in the initial CT simulation scan and all subsequent CBCT images.

Clip displacement (i.e. inter- and intrafractional movement of the mass) was assessed using Image Fusion from the Pinnacle 9.2 TPS. Cone beam CT images from each treatment day were fused with the initial planning CT for bony registration. Displacement was calculated by comparing the coordinates of the geometric centers of the contoured clip volume (i.e. the GTV) of the initial planning CT versus daily CBCTs. The average interfraction movement of the clips was 0.69 cm (ranging 0.51-0.98 cm) from initial planning (Table 1).

**Table 1:** **Interfractional displacement of surgical clips on treatment day from planning**

**Treatment day**	**Interfractional movement (cm)**
0 (Planning)	--
1	0.98
2	0.51
3	0.55
4	0.61
5	0.64

CBCT images were taken at planning and before each day of treatment. Images were fused with the planning CT image after bony registration. Displacement of the clips between the each day and planning were calculated. Pre- and post-shift displacements are measured from the central coordinates of the GTV.

On days 4 and 5 of treatment, the patient required additional repositioning/shifting to center the PTV onto the target. Intrafractional movement (i.e. the difference between daily pre- and post-shift movement) of the patient produced a 0.24 cm and 0.41 cm displacement of the target on days 4 and 5, respectively (Table 2). The clips had the greatest mean magnitude of displacement in the left-right dimension (0.83 cm), followed by superior-inferior (0.51 cm) and antero-posterior (0.44 cm) dimensions (data not shown). As inter- and intra-fractional displacement is not yet well understood for tumours located within the colon, PTV margins were maintained at 1 cm throughout the treatment course to ensure adequate coverage.

**Table 2:** **Intrafractional movement of the GTV on Day 4 & 5**

**Treatment day**	**Pre-shift displacement (cm)**	**Post-shift displacement (cm)**	**Change in displacement (cm)**
4	0.61	0.44	0.21
5	0.64	0.35	0.41

The patient was repositioned on days 4 and 5 after initial CBCT to improve GTV alignment and conform to PTV margins. The change in magnitude of intrafractional displacement is shown.

### Clinical follow-up

The patient tolerated the palliative course of radiation treatment well with no gastrointestinal or genitourinary toxicity observed. Lower gastrointestinal bleeding stopped within 4 days of completing radiation therapy and post-treatment HgB level was 95 mg/L; no additional transfusions were required. At six-week follow-up, the patient did not have any recurrent lower GI bleeding and HgB had increased to 103 mg/L. At a later date, the patient was admitted to hospital with thromboembolic stroke and died from complications.

## Discussion

Given the complicated and unfortunate course of disease for this patient, palliative management was recommended to control lower GI bleeding, avoid further transfusions, and improve quality of life. Rare abdominal and pelvic metastasis of papillary thyroid cancer presented a challenge for management, and only a handful of reports have been published [4-6]. One report documented a 19-year old girl with a rare cribriform-morular variant of PTC, associated with familial adenomatous polyposis syndrome [4]. Another report described Hurthle cell thyroid carcinoma metastatic to the sigmoid colon with radioactive iodine resistance [6]. However, these studies were primarily treated with surgery and did not utilize external beam radiation therapy for the abdominal and pelvic metastases. As noted, surgery for this patient was contraindicated due to the extent of metastatic disease and comorbidities.

Abdominal organ and tumor movements are influenced by respiration and bladder filling status, leading to irregular changes in conformity [9]. Abbas et al. reviewed multiple motion management strategies to control abdominal organ movement for radiation therapy, including motion control/suppression methods and respiratory gating during treatment; however, movement of the intestines may present additional challenges due to the relatively unfixed nature of the organs compared to other abdominal organs [9].

Irradiation of the abdomen and pelvis may cause acute toxicities including nausea, vomiting and diarrhea. For palliative dose regimens such as 20 Gy in 5 fractions the reported rates of nausea and/or vomiting range between 30-50% [10-12]. Biological models and clinical studies suggest that the volume of small bowel loops receiving 15 Gy or more (V15) may be an important factor. A threshold of V15 > 120 cm^3^ is associated with >10% risk of grade 3 or higher acute toxicity [13-15]. We retrospectively contoured the loops of small bowel and evaluated the V15 for the radiotherapy plans with and without fiducial markers. The preliminary radiation plan resulted in a V15 of 328 cm^3^ which would have exposed the patient to a high risk of severe acute small bowel toxicity[13]. The plan based on surgical clips for target delineation yielded close to 50% reduction in the small bowel dose, with a V15 of 176 cm^3^, suggesting a potential decrease in the risk of severe toxicity.

Inter- and intrafractional tumor movement have been assessed with regards to lung, pancreatic, prostatic, vaginal and hepatic cancers, but not lesions in the colon [16-20]. Recent case studies have also employed the use of surgical clips as fiducial markers in radiosensitive intra-abdominal lymphomas in the duodenum and colon but did not report on tumour movement and adequacy of PTV margins [7,9]. Other studies have assessed the use of fiducial markers and daily CBCT registration in bladder, pancreas and prostate, which have found significant discrepancies between interfractional fiducial marker location and bony anatomy [21-23]. Despite the improved tumor visualization with fiducial markers, proper organ motion data must be assessed to accurately delineate the target margins to avoid inappropriate dosing [8]. In this case, CBCT was used to first ensure bony matching and then to confirm tumour coverage. The clip displacements after bony registration ranged between 0.21-0.98 cm, therefore additional shifts were not required and the clinical impact was limited. In future studies, clip displacement might be used to evaluate systematic errors and reduce PTV margins.

## Conclusion

Here we present a rare case of papillary thyroid cancer metastasis to the colon treated with a palliative dose of IGRT to control lower GI bleeding. Surgical clips were used as fiducial markers with a volumetric expansion of 1 cm for the CTV and 1 cm for the PTV. The main benefit of the fiducial markers in this case was accurate delineation of the GTV which allowed for smaller target volumes. Using daily CBCT, the interfraction and intrafraction clip movements were measured to be less than 1 cm which was within the PTV margin chosen for this case.

This study demonstrates the clinical efficacy of palliative IGRT for a colon tumor using surgical clips as fiducial markers to enhance target localization and verify daily treatment position. The radiopaque surgical clips provide a reliable marker for visual verification of a mobile and otherwise imperceptible lesion.

## Consent

Informed consent was obtained from the patient and next of kin for publication of this case report and any accompanying images.

## Abbreviations

PTC: Papillary thyroid cancer
IGRT: Image-guided radiation therapy
GTV: Gross tumor volume
PTV: Planning target volume
CBCT: Cone beam computed tomography
GERD: Gastroesophageal reflux disease
TPS: Treatment planning system
